# Predictive modeling of gene mutations for the survival outcomes of epithelial ovarian cancer patients

**DOI:** 10.1371/journal.pone.0305273

**Published:** 2024-07-08

**Authors:** Mirielle C. Ma, Ethan S. Lavi, Gary Altwerger, Z. Ping Lin, Elena S. Ratner

**Affiliations:** Department of Obstetrics, Gynecology, and Reproductive Sciences, Yale University School of Medicine, New Haven, Connecticut, United States of America; CNR, ITALY

## Abstract

Epithelial ovarian cancer (EOC) has a low overall survival rate, largely due to frequent recurrence and acquiring resistance to platinum-based chemotherapy. EOC with homologous recombination (HR) deficiency has increased sensitivity to platinum-based chemotherapy because platinum-induced DNA damage cannot be repaired. Mutations in genes involved in the HR pathway are thought to be strongly correlated with favorable response to treatment. Patients with these mutations have better prognosis and an improved survival rate. On the other hand, mutations in non-HR genes in EOC are associated with increased chemoresistance and poorer prognosis. For this reason, accurate predictions in response to treatment and overall survival remain challenging. Thus, analyses of 360 EOC cases on NCI’s The Cancer Genome Atlas (TCGA) program were conducted to identify novel gene mutation signatures that were strongly correlated with overall survival. We found that a considerable portion of EOC cases exhibited multiple and overlapping mutations in a panel of 31 genes. Using logistical regression modeling on mutational profiles and patient survival data from TCGA, we determined whether specific sets of deleterious gene mutations in EOC patients had impacts on patient survival. Our results showed that six genes that were strongly correlated with an increased survival time are BRCA1, NBN, BRIP1, RAD50, PTEN, and PMS2. In addition, our analysis shows that six genes that were strongly correlated with a decreased survival time are FANCE, FOXM1, KRAS, FANCD2, TTN, and CSMD3. Furthermore, Kaplan-Meier survival analysis of 360 patients stratified by these positive and negative gene mutation signatures corroborated that our regression model outperformed the conventional HR genes-based classification and prediction of survival outcomes. Collectively, our findings suggest that EOC exhibits unique mutation signatures beyond HR gene mutations. Our approach can identify a novel panel of gene mutations that helps improve the prediction of treatment outcomes and overall survival for EOC patients.

## Introduction

Epithelial ovarian cancer (EOC) is cancer derived from the outer lining of the ovaries, the main female reproductive organ [1]. It is the second most common gynecologic cancer in the United States, amounting to about 14,000 deaths per year with a 5-year overall survival rate of 47% [2]. High grade serous EOC is the most common form of ovarian cancer, accounting for about 70% of cases [3]. The high mortality rate is attributable to late-stage diagnosis that reduces the clinical response to the treatment. Furthermore, due to a high rate of recurrence it is common for patients to develop resistance to platinum-based chemotherapy and possibly poly ADP ribose polymerase (PARP) inhibitor therapy [4, 5]. Eventually patients succumb to the disease because chemo-resistant EOC cells no longer respond to most treatment modalities [6].

Using data from NCI’s Cancer Genome Atlas (TCGA) program, we aimed to survey the landscape of gain-of-function mutations in oncogenes and loss-of function gene mutations in tumor suppressor genes for the responses of EOC to platinum-based chemotherapy in EOC patients. We analyzed 360 cases of EOC to identify common gene mutations that could potentially predict clinical responses to treatment and survival outcomes in patients. Finding patterns in gene mutations intimately associated with therapeutic responses can also help decide appropriate therapy to improve long-term survival rates [7]. Homologous recombination (HR) repair deficiency is the most prominent and characterized EOC phenotype that indicate increased sensitivity to DNA damage therapies [8, 9]. EOC cells that have faulty HR repair are not able to fix DNA double strand breaks (DSBs) induced by platinum-based chemotherapy or PARP inhibitors [10]. However, it remains largely unknown that other gene mutations play a role in EOC sensitivity to platinum-based chemotherapy and other cancer therapeutics.

Many EOC patients have a high mutational load with combinatory gene mutations that have positive and negative effects on survival [11]. Taking account of all the effects of these gene mutations, we established a statistical model that generated a prediction of whether a patient had an increased or decreased survival. Our model offers a new perspective on the relationship between gene mutation profiles and patient survival outcomes. It would also provide potential targets to spark future investigations and development of therapeutic interventions.

## Materials and methods

### Data acquisition

The data from the public database The Cancer Genome Atlas (TCGA) were acquired through the cancer.gov website since June of 2020. The database is part of NCI’s Genomic Data Commons (GDC) portal. Within the TCGA database, ovary was selected as the primary site in the TCGA-OV project. A total of 582 EOC cases were collected. We chose 38 genes for the study based on the frequency of mutation as well as the role that a gene plays in DNA damage repair pathways such as HR repair and mismatch repair genes. Given that not all mutations have negative effects on gene functions, we selected 360 patients who had deleterious gene mutations identified by one or more predictive algorithms: Variant Effect Predictor (VEP), Sorting Intolerant from Tolerant (SIFT), and Polymorphism Phenotyping (PolyPhen). Deleterious mutations were classified as VEP Impact high or moderate, SIFT Impact deleterious or deleterious low confidence, and PolyPhen probably or possibly damaging. Because 7 genes showed perfect separation that hindered logistic regression analysis, we analyzed 31 common gene mutations in relation to the survival outcomes of these EOC patients. Using the same approach, we additionally analyzed 25 deleterious mutations in relation to the survival outcomes of 437 uterine cancer patients (corpus uteri in the TCGA-UCEC database), as well as 21 deleterious mutations in relation to the survival outcomes of 176 cervical cancer patients (from the TCGA-CESC database). The data were entered into a Microsoft Excel spreadsheet as categorical variables (yes vs no) for all gene mutations and survival outcomes of patients (days to death and overall survival). Mutations were defined as yes and no–if at least one deleterious mutation was present in the gene, then the gene was categorized as mutated. The data were also counted for the mutation frequency of each gene. The datasets of survival outcomes and gene mutations in EOC, uterine cancer, and cervical cancer patients are listed in the Zenodo repository [12].

### Logistic regression modeling

Logistic regression was used to model the pattern of 31 gene mutations in 360 EOC patients in relation to their survival outcomes. Uterine and cervical cancer patients were analyzed in a similar manner to EOC patients. The presence of a gene mutation was set as “1” and the absence of a gene mutation is set as “0”. An alive patient was set as “1” and a deceased patient was set as “0”. The analysis was performed using the Prism 9 software (GraphPad). The survival outcome was the logit function [Log (p/(1-p), where p is probability] of all 31 gene mutations:

$$\begin{array}{ccc} Log\left(\frac{p}{1-p}\right) & = & \beta 0(\text{intercept})+\beta 1\times \text{TP53}+\beta 2\times \text{BRCA1}+\beta 3\times \text{BRCA}2+\beta 4\times \text{KMT2C}\\ & & +\beta 5\times \text{CDK12}+\beta 6\times \text{CSMD3}+\beta 7\times \text{TTN}+\beta 8\times \text{MUC16}+\beta 9\times \text{NF1}\\ & & +\beta 10\times \text{PALB2}+\beta 11\times \text{RB1}+\beta 12\times \text{PTEN}+\beta 13\times \text{FOXM1}+\beta 14\times \text{BRIP1}\\ & & +\beta 15\times \text{KRAS}+\beta 16\times \text{RASA1}+\beta 17\times \text{CHEK2}+\beta 18\times \text{RAD50}\\ & & +\beta 19\times \text{MRE11A}+\beta 20\times \text{NBN}+\beta 21\times \text{FANCE}+\beta 22\times \text{FANCD}2\\ & & +\beta 23\times \text{ATR}+\beta 24\times \text{ATM}+\beta 25\times \text{MSH2}+\beta 26\times \text{PMS2}+\beta 27\times \text{MLH1}\\ & & +\beta 28\times \text{FAT1}+\beta 29\times \text{FAT2}+\beta 30\times \text{FAT3}+\beta 31\times \text{FAT4} \end{array}$$

β1–31 was the estimate for each gene mutation. p = 0.5 was used as the cutoff; p > 0.5 predicted the alive outcome whereas p < 0.5 predicted the deceased outcome for a patient.

To evaluate the contribution of individual gene mutation to the survival outcome, the estimates of all 31 genes were ranked. Positive estimate values contributed to an increase in the probability of survival whereas negative estimate values contributed to a decrease in the probability of survival. Z-score, p-value, and odd ratio for each estimate was also shown to evaluate the contribution of the gene mutation to the survival outcomes of patients.

### Classification metrics and ROC curve

Classification metrics was used to evaluate the performance of the logistic regression model by comparing predicted survival outcomes with actual survival outcomes.

True positive (TP): the model predicted the alive outcome correctly. True negative (TN): the model predicted the dead outcome correctly. False positive (FP): the model predicted the alive outcome incorrectly. False negative (FN): the model predicted the dead outcome incorrectly. Accuracy = (TP+TN)/(TP+TN+FP+FN) was defined as the overall correctness of the model’s predictions. Precision = TP/(TP+FP) represented the accuracy of prediction. Recall = TP/(TP+FN) quantified the accuracy of prediction. The receiver operating characteristic (ROC) curve and the area under the ROC curve used to assess the performance of the logistic regression model were generated by the Prism software.

### Kaplan-meier survival analysis

Kaplan-Meier survival analysis was conducted using the Prism 6 software (GraphPad). 360 EOC patients were grouped by the presence and absence of a gene mutation spectrum, such as 6 positive genes, 6 negative genes, or HR-deficient genes. The survival times of patients were used to define the survival endpoints. If patients were still alive, 5,000 days were entered as the endpoints. The median survival times were determined. The Log-rank (Mantel-Cox) test was used to determine statistical significance between groups.

### Pearson correlation analysis

Pearson correlation analysis was performed with the same dataset for logistic regression modeling, using NCSS 2024 software (NCSS). The alive outcome and 31 gene mutations were input as variables to identify pair-wise correlations and generate a matrix of the heat map and hierarchical clustering of variables. The scatter plot of Eigenvectors of Pearson correlations was generated to indicate the relatedness of the alive outcome to 31 genes.

## Results

Using the data of 360 patients on TCGA, we analyzed 31 genes that were frequently mutated in EOC patients. After analyzing the mutation frequency of each gene and its corresponding survival rate, we listed mutated genes and showed their impacts on the survival of patients. The averaged overall survival rate for all 360 EOC cases was 44.2%. TP53 was the most frequently mutated gene at 96.4% and with a 44.1% survival rate (**Table 1**). TTN was the second frequently mutated gene at 46.9% and with a 37.9% survival rate. BRCA1 mutation was associated with the highest survival rate of 72.7% and a 6.1% frequency. RAD50, MRE11A, and NBN mutations also had a high survival rate of 66.7% but a low (0.8%) frequency. On the other hand, the FANCE, FOXM1, and KRAS mutations caused the lowest survival rate of 14–25% at relatively lower (1.1–2.5%) frequencies.

**Table 1:** Frequent gene mutations and survival rates in 360 EOC patients.

	Mutated EOC	% Mutated	Alive with Mutation	% Overall Survival
**TP53**	347	96.4	153	44.1
**TTN**	169	46.9	64	37.9
**MUC16**	52	14.4	21	40.4
**CSMD3**	41	11.4	13	31.7
**KMT2C**	39	10.8	20	51.3
**FAT3**	39	10.8	18	46.2
**NF1**	28	7.8	15	53.6
**FAT4**	28	7.8	14	50.0
**FAT1**	23	6.4	9	39.1
**BRCA1**	22	6.1	16	72.7
**CDK12**	20	5.6	8	40.0
**FAT2**	20	5.6	10	50.0
**RB1**	18	5.0	8	44.4
**BRCA2**	17	4.7	7	41.2
**ATM**	13	3.6	5	38.5
**PTEN**	10	2.8	5	50.0
**ATR**	10	2.8	5	50.0
**PALB2**	9	2.5	4	44.4
**FOXM1**	9	2.5	2	22.2
**FANCD2**	9	2.5	3	33.3
**FANCE**	7	1.9	1	14.3
**MSH2**	5	1.4	2	40.0
**MLH1**	5	1.4	2	40.0
**BRIP1**	4	1.1	2	50.0
**KRAS**	4	1.1	1	25.0
**RASA1**	4	1.1	2	50.0
**PMS2**	4	1.1	2	50.0
**CHEK2**	3	0.8	1	33.3
**RAD50**	3	0.8	2	66.7
**MRE11A**	3	0.8	2	66.7
**NBN**	3	0.8	2	66.7

The ranking of commonly mutated genes and associated survival rates identified by TCGA is shown. 360 cases of high-grade serous EOC cases were analyzed for their number of mutations, mutation frequency, and overall 10-year survival rate.

**Fig 1:**
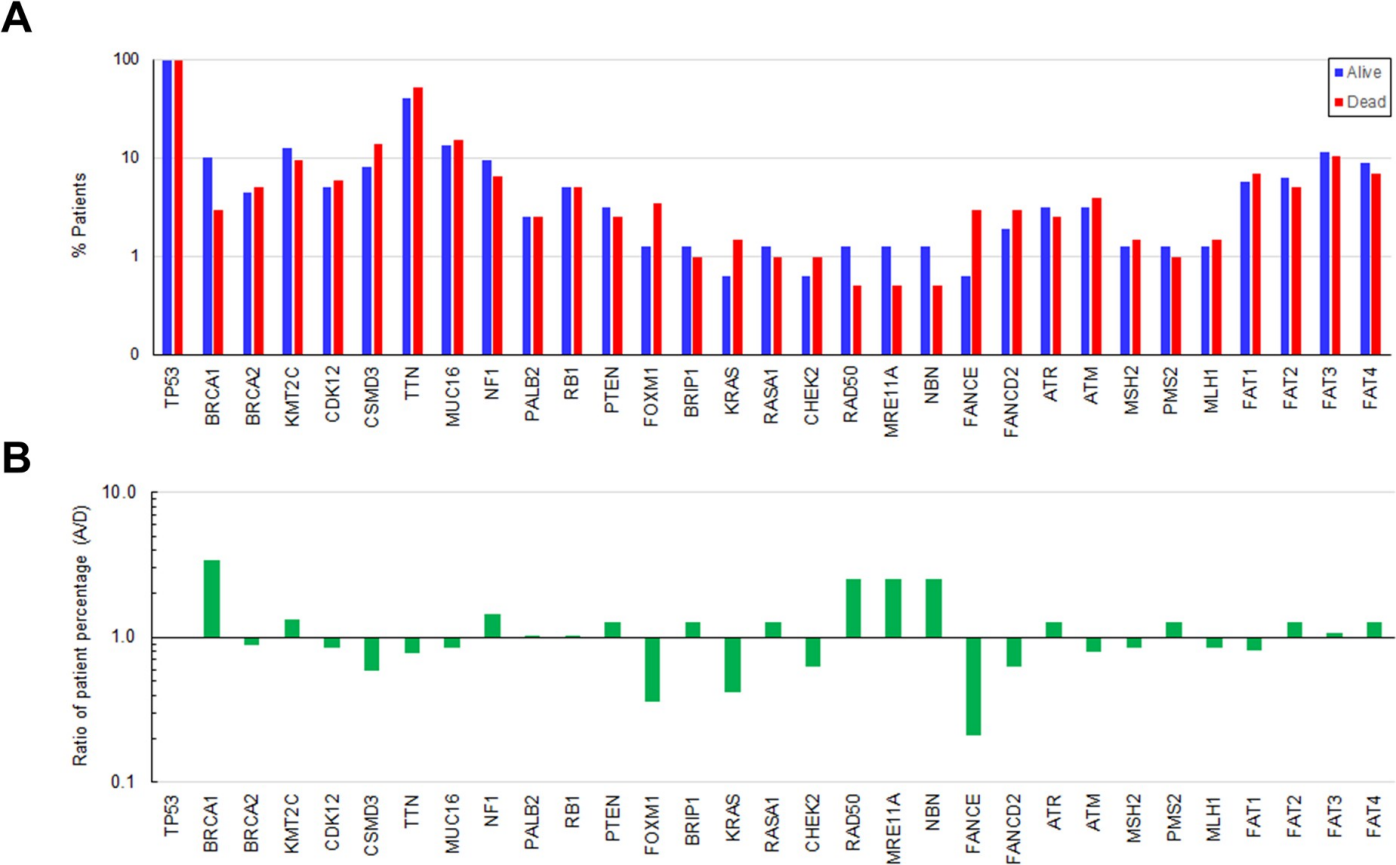
Comparison of gene mutations between alive and deceased EOC patients. A) The percentages of alive and deceased patients that contained each of 31 gene mutations were determined. 159 patients were alive, and 201 patients were deceased. B) The ratios of the percentages of alive to deceased patients were calculated. The bars indicate a fold change in the percentage of alive patients compared with deceased patients in each gene.

To gain the insights into the landscape of gene mutations in 360 patients in relation to survival outcomes, we compared the percentage of alive and deceased patients in each of 31 genes. The ratios of the percentage of alive to deceased patients were also calculated. The results show that there was a greater percentage of alive patients that had mutations in genes including BRCA1, RAD50, MRE11A, and NBN (**Fig 1**). In contrast, there was a higher percentage of deceased patients that had mutations in genes including FOXM1, KRAS, FANCE, and FANCD2.

Because a vast majority of 360 EOC patients exhibited multiple mutations (2–10 mutations) of these 31 genes analyzed, we determined whether there were patterns of combinatory gene mutations that would better correlate with and predict the survival outcomes of patients. We performed the logistic regression analysis to model the survival outcomes (alive and deceased) as a function of the combination of 31 gene mutations in 360 patients. The estimates of 31 genes for the logistic regression model were obtained and ranked to evaluate the positive and negative effects of gene mutations on survival. Of the 31 mutations, 16 genes with positive estimates showed correlation with improved survival, and 15 genes with negative estimates were correlated with decreased survival (**Table 2**). Despite that only the estimates of BRCA1 and TTN were statistically significant, top-ranking positive and negative genes were highly correlated with highest and lowest odd ratios, respectively, indicative of their strongest impacts on survival outcomes. Mutations that cause HR deficiency were mostly correlated with increased survival, while FA genes were all correlated with decreased survival. Based on the ranking, BRCA1 mutation was the most positive predictor of increased survival whereas FANCE mutation was the most negative predictor of decreased survival. The area under the ROC curve for this model is 0.651 ± 0.029 (95% CI, 0.5930–0.7080) (**S1A Fig**). The overall accuracy of this model for EOC was 64.2%. The other classification metrics are shown in **Table 3**.

**Table 2:** Logistic regression analysis of 31 gene mutations and survival outcomes in 360 EOC patients.

Variable	Estimate	Estimate 95% CI	|Z|	p	Odd Ratio	Odd Ratio 95% CI
**BRCA1**	1.347 ± 0.530	0.357 to 2.469	2.541	0.011	3.84	1.429 to 11.81
**NBN**	1.116 ± 1.337	-1.404 to 4.354	0.835	0.404	3.05	0.246 to 77.77
**BRIP1**	0.733 ± 1.152	-1.626 to 3.173	0.636	0.525	2.08	0.197 to 23.88
**RAD50**	0.719 ± 1.316	-1.832 to 3.889	0.546	0.585	2.05	0.160 to 48.88
**PTEN**	0.621 ± 0.697	-0.769 to 2.035	0.892	0.373	1.86	0.463 to 7.651
**PMS2**	0.577 ± 1.082	-1.664 to 2.841	0.534	0.594	1.78	0.189 to 17.14
**FAT2**	0.513 ± 0.548	-0.567 to 1.610	0.937	0.349	1.67	0.567 to 5.001
**NF1**	0.343 ± 0.429	-0.499 to 1.197	0.799	0.425	1.41	0.607 to 3.311
**MRE11A**	0.307 ± 1.372	-2.346 to 3.559	0.224	0.823	1.36	0.0957 to 35.12
**PALB2**	0.305 ± 0.757	-1.236 to 1.824	0.403	0.687	1.36	0.291 to 6.195
**FAT3**	0.305 ± 0.375	-0.435 to 1.044	0.814	0.415	1.36	0.648 to 2.839
**FAT4**	0.279 ± 0.431	-0.573 to 1.133	0.646	0.519	1.32	0.564 to 3.105
**KMT2C**	0.260 ± 0.389	-0.507 to 1.026	0.670	0.503	1.30	0.602 to 2.791
**CHEK2**	0.162 ± 1.406	-3.073 to 3.162	0.115	0.909	1.18	0.0463 to 23.61
**ATR**	0.157 ± 0.723	-1.315 to 1.592	0.217	0.828	1.17	0.269 to 4.913
**RB1**	0.003 ± 0.524	-1.051 to 1.032	0.005	0.996	1.00	0.350 to 2.805
**CDK12**	-0.010 ± 0.506	-1.038 to 0.973	0.019	0.985	0.99	0.354 to 2.646
**MSH2**	-0.024 ± 1.007	-2.202 to 1.953	0.024	0.981	0.98	0.111 to 7.053
**RASA1**	-0.028 ± 1.187	-2.439 to 2.459	0.023	0.981	0.97	0.0873 to 11.69
**TP53**	-0.036 ± 0.693	-1.429 to 1.343	0.051	0.959	0.97	0.240 to 3.831
**MUC16**	-0.037 ± 0.334	-0.700 to 0.616	0.109	0.913	0.96	0.497 to 1.852
**BRCA2**	-0.038 ± 0.533	-1.126 to 0.996	0.071	0.943	0.96	0.324 to 2.707
**Intercept**	-0.054 ± 0.709	-1.462 to 1.370	0.076	0.940	0.95	0.232 to 3.937
**ATM**	-0.076 ± 0.632	-1.389 to 1.144	0.121	0.904	0.93	0.249 to 3.139
**MLH1**	-0.144 ± 1.061	-2.491 to 1.895	0.136	0.892	0.87	0.0829 to 6.656
**FAT1**	-0.222 ± 0.492	-1.221 to 0.731	0.452	0.652	0.80	0.295 to 2.076
**CSMD3**	-0.463 ± 0.381	-1.236 to 0.268	1.217	0.224	0.63	0.290 to 1.307
**TTN**	-0.581 ± 0.238	-1.051 to -0.118	2.444	0.015	0.56	0.349 to 0.889
**FANCD2**	-0.738 ± 0.813	-2.487 to 0.804	0.908	0.364	0.48	0.0831 to 2.235
**KRAS**	-1.035 ± 1.292	-4.209 to 1.316	0.801	0.423	0.36	0.0149 to 3.728
**FOXM1**	-1.392 ± 0.945	-3.525 to 0.321	1.472	0.141	0.25	0.0295 to 1.378
**FANCE**	-1.564 ± 1.150	-4.585 to 0.365	1.360	0.174	0.21	0.0102 to 1.440

The table ranks the estimates for each gene mutation variable, 95% confidence interval (CI) for estimates, Z-score, p-value, odd ratio, and 95% CI for odd ratio in the logistic regression model. The positive estimates of gene mutations were collectively associated with improved survival whereas the negative estimates of gene mutations collectively contributed to poor survival.

**Table 3:** Classification metrics for evaluation of the predictive model for the survival outcomes of 360 EOC patients.

	Predicted dead (number of patients)	Predicted alive (number of patients)	Accuracy (%)	Precision (%)	Recall (%)
**Actual dead**	176	25	64.2	68.8	34.6
**Actual alive**	104	55			

**Fig 2:**
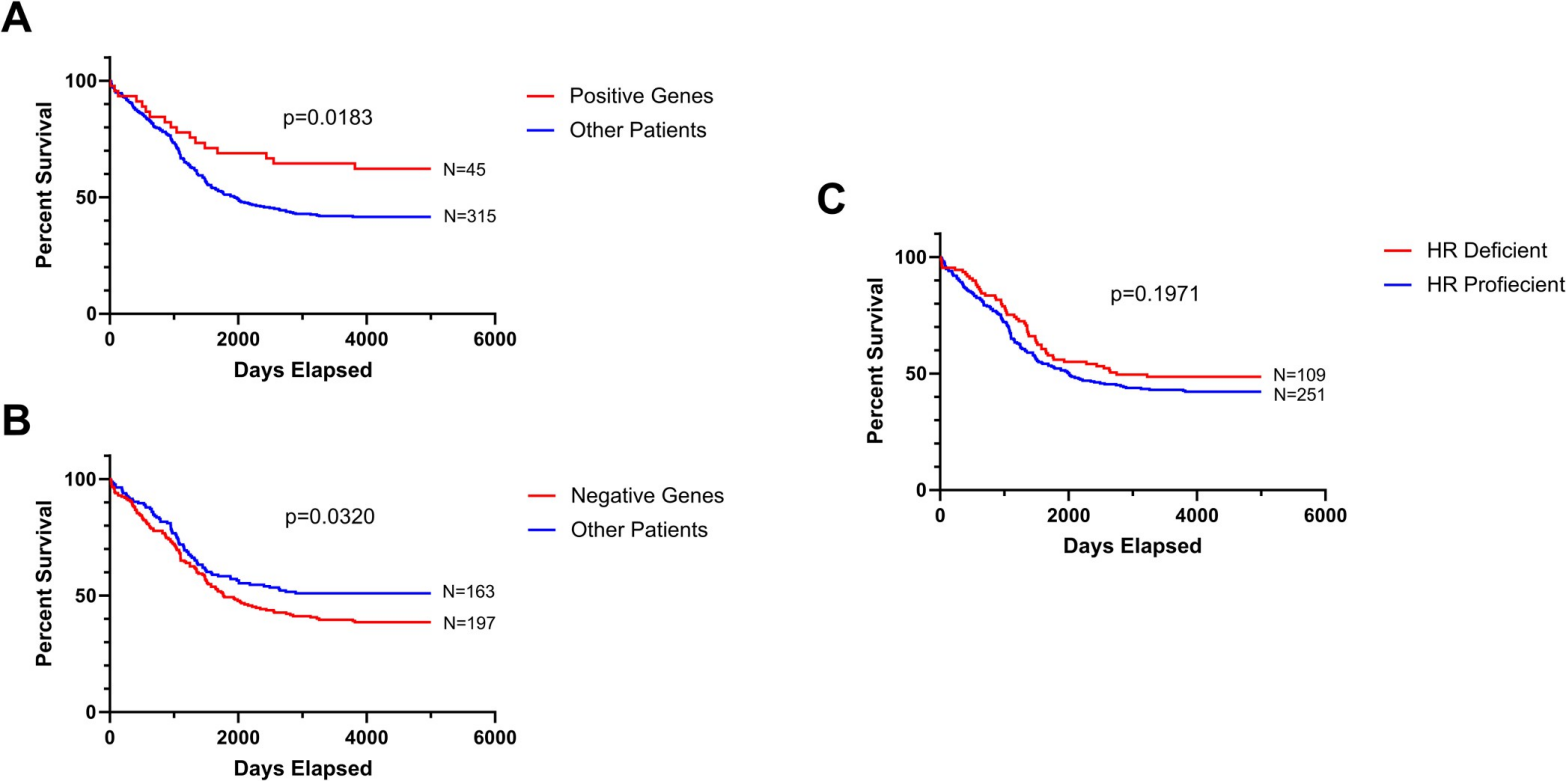
Kaplan-meier survival analysis of 360 EOC patients. Kaplan-Meier survival analysis was performed on the basis of stratified patient populations containing mutations in positive genes (BRCA1, NBN, BRIP1, RAD50, PTEN, and PMS2) (A), negative genes (FANCE, FOXM1, KRAS, FANCD2, TTN, and CSMD3) (B), and HR-deficient genes (BRCA1, BRCA2, ATM, ATR, BRIP1, CDK12, CHEK2, FANCD2, FANCE, MRE11, NBN, PALB2, and RAD50) (C). The number of patients stratified (N) was also shown.

Based on our logistic regression model, we performed Kaplan-Meier survival analysis of 360 patients stratified by positive or negative gene mutations. The group of patients containing any of the top 6 genes (BRCA1, NBN, BRIP1, RAD50, PTEN, and PMS2) correlated with increased survival (positive gene group) was compared with the group of all other patients lacking these mutations. The Kaplan-Meier survival curves show that the positive gene group exhibited a significant longer survival time than the group of other patients (p = 0.0183) (**Fig 2A**). The median survival times were >10 years for the positive gene group and 5.3 years for the other patient group. Likewise, the group of patients containing any of the top 6 genes (FANCE, FOXM1, KRAS, FANCD2, TTN, and CSMD3) correlated with decreased survival (negative gene group) was compared with the group of all other patients lacking these mutations. The negative gene group displayed a significant shorter survival time than the group of other patients (p = 0.0320) (**Fig 2B**). The median survival times were 4.8 years for the negative gene group and >10 years for the other patient group.

To substantiate our findings, we further conducted the Kaplan-Meier survival analysis of 360 patients grouped by the conventional definition of HR status [13]. Patients that contained mutations in BRCA1, BRCA2, ATM, ATR, BRIP1, CDK12, CHEK2, FANCD2, FANCE, MRE11, NBN, PALB2, and RAD50 were categorized as the HR deficient group. Patients that lacked these mutations were categorized as the HR proficient group. The analysis indicates that the survival times of HR-deficient and HR-proficient groups were not statistically different (p = 0.1971)(**Fig 2C**). The median survival times of HR-deficient and HR-proficient groups were 7.5 and 5.5 years, respectively.

To further validate this predictive model with other gynecologic cancers, we performed similar analyses on the dataset of 437 uterine cancer patients and 23 gene mutations from TCGA. Based on the ranking of estimates and odd ratio, ATM, FANCD2, BRCA1, MSH6, CHEK2, and MUC16 were identified as top 6 positive genes while CDK12, BRIP1, FAT3, TP53, NF1, and PMS2 were identified as top 6 negative genes (**S1 Table**). The area under the ROC curve for the uterine cancer model is 0.723 ± 0.032 (95% CI, 0.660–0.787) (**S1B Fig**). The overall accuracy of this model for uterine cancer was 81.5%. The other classification metrics are shown in **S2 Table**. Similar to EOC, we performed Kaplan-Meier survival analysis of 437 uterine cancer patients stratified by positive or negative gene mutations. The result showed that patients with positive genes had a significantly better survival outcome (p = 0.0179) and patients with negative genes had a significantly worse survival outcome (p = 0.0020) than their respective other patient groups (**S2A and S2B Fig**).

Moreover, we analyzed the dataset of 174 cervical cancer patients and 21 gene mutations from TCGA. CSMD3, FAT1, BRCA1, PALB2, NF1, and RB1 were identified as top 6 positive genes. MLH1, FANCD2, BRCA2, CDK12, TP53, and FAT3 were identified as top 6 negative genes. (**S3 Table**). The area under the ROC curve for the cervical cancer model is 0.750 ± 0.045 (95% CI, 0.662–0.837) (**S1C Fig**). The overall accuracy of this model for cervical cancer was 79.3%. The other classification metrics are shown in **S4 Table**. Kaplan-Meier survival analysis of 174 cervical cancer patients stratified by these positive or negative gene mutations. The result was consistent with those of EOC and uterine cancer showing that patients with positive genes had a significantly better survival outcome (p = 0.0040) and patients with negative genes had a significantly worse survival outcome (p = 0.0088) than their respective other patient groups (**S2C and S2D Fig**).

Collectively, these results suggest that our logistic regression model identifies gene mutation profiles predictive of the survival outcomes of EOC patients and outperforms traditional HR status-based approaches. The same approach is also applicable to uterine and cervical cancer patients.

**Fig 3:**
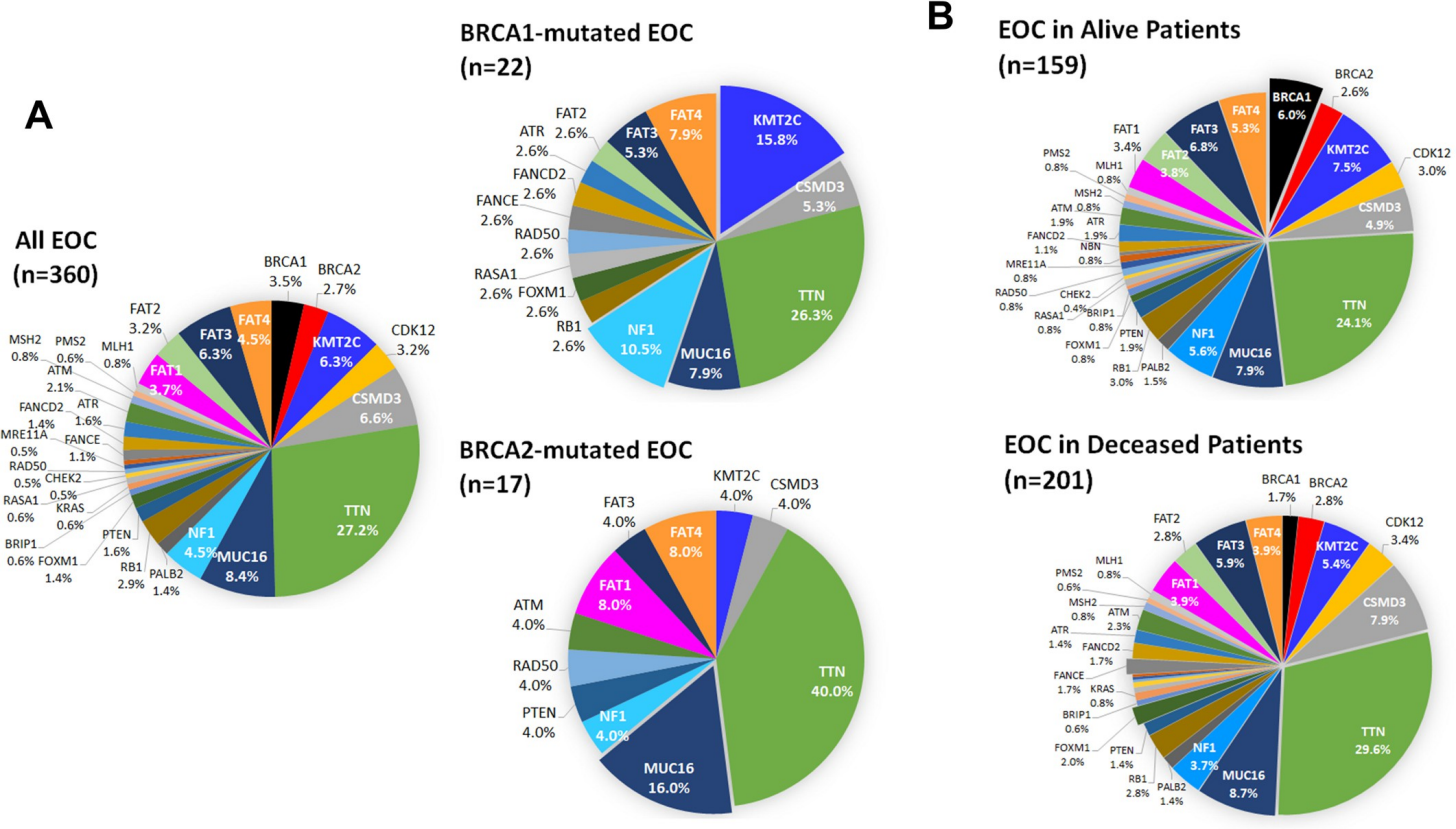
Mutation frequency in EOC populations. A) Gene mutation spectrums of BRCA1- and BRCA2-mutated EOC. The percentages of 30 gene mutations are displayed in all, BRCA1-mutated, and BRCA2-mutated EOC cases. B) Gene mutation spectrums of EOC in alive and deceased patients. The percentages of 30 gene mutations are displayed for alive and deceased EOC patients. TP53 mutations are not included in the pie charts because these mutations are present in 96.4% of patients.

The gene mutation spectrums of BRCA-mutated EOC in 39 patients were analyzed and compared with that of all EOC in 360 patients. The percentages of 30 gene mutations were presented in the pie charts except for TP53 because TP53 mutations were found in 96.4% of EOC cases. BRCA1- and BRCA2-mutated EOC exhibited discernible mutation patterns (**Fig 3A**). KMT2C and NF1 mutations considerably increased in BRCA1-mutated EOC. In contrast, TTN and MUC16 mutations substantially elevated and accounted for 56% of all mutations analyzed in BRCA2-mutated EOC. FAT1 mutation was absent in BRCA1-mutated EOC while FAT1 mutation appeared to increase in BRCA2-mutated EOC. These results suggest that BRCA1 mutation clusters with KMT2C and NF1 mutations and BRCA2 mutation clusters with TTN and MUC16 mutations in EOC.

The gene mutation spectrum of EOC were comparatively analyzed in alive and deceased patient populations. BRCA1 mutation was substantially greater in alive patients than in deceased patients (**Fig 3B**). In contrast, FANCE mutation was noticeably enriched in deceased patients compared with alive patients. These finding suggest that BRCA1 mutation is correlated with an improved survival whereas FANCE mutation is associated with a decreased survival in patients.

**Fig 4:**
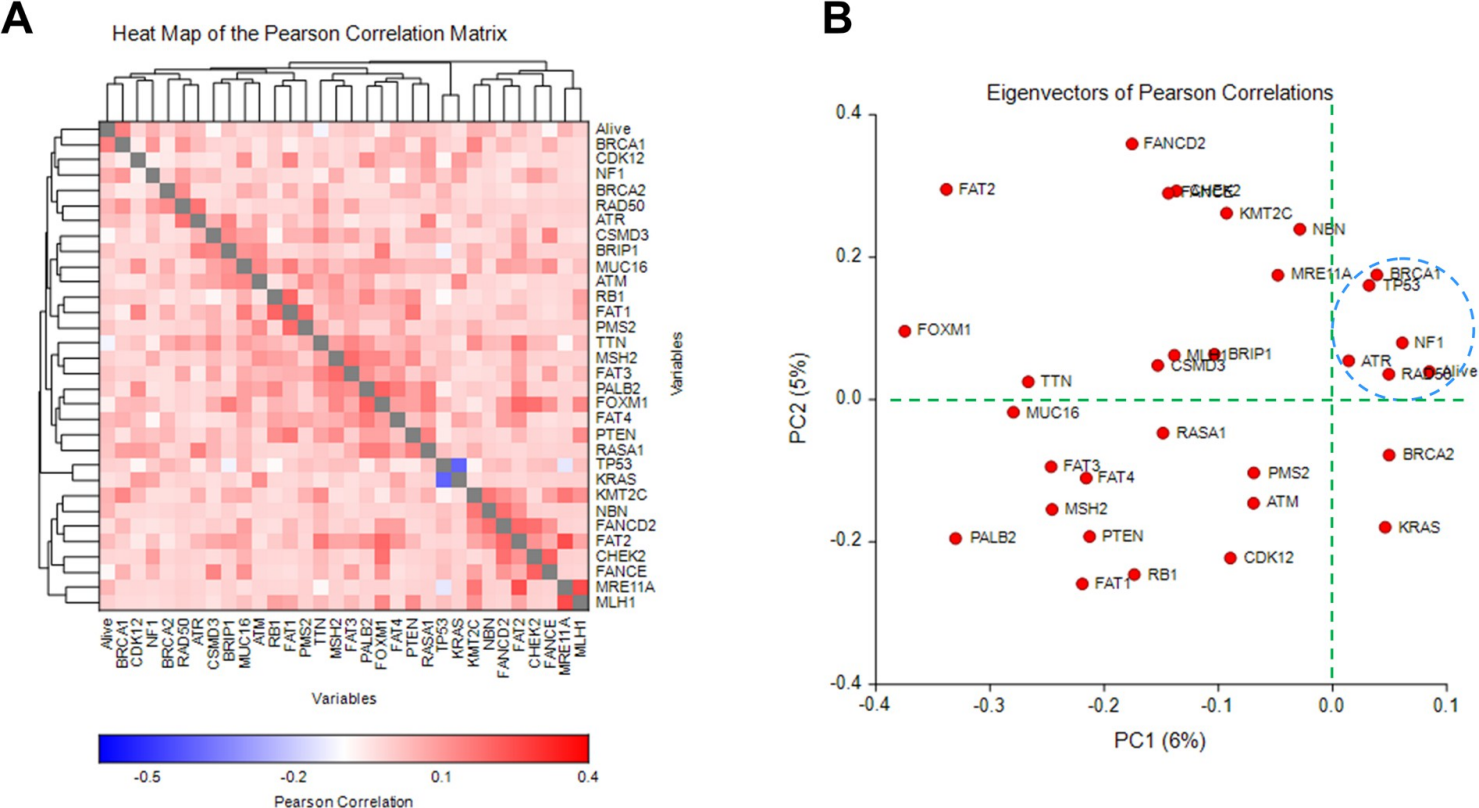
Heat map matrix and Eigenvectors of Pearson correlation analysis of 31 gene mutations and the alive outcome in 360 EOC patients. Pearson correlation analysis was performed to make pair-wise comparisons to identify correlations among variables (alive outcome and 31 gene mutation). A) The correlation matrix is displayed as a heatmap. The correlations among variables are sorted to show hierarchical clustering. B) The Eigenvectors of Pearson correlations are shown to cluster variables and identify their relatedness on the PC1 versus PC2 scatter plot. The blue dash circle highlights the close relationship among the alive outcome and gene mutations.

To further corroborate the findings from our logistic regression model, survival analysis, and gene mutation spectrums, we performed correlation matrix analysis on the alive outcomes and 31 gene mutations in 360 EOC patients. The results of the Pearson correlation matrix show that the BRCA1 mutation was the most positively associated with the alive outcome of patients (**Fig 4A**). NF1, RAD50, ATR, and many gene mutations also exhibited a positive correlation with the alive outcome to a various degree. By contrast, TTN, FANCE, CSMD3, and FOXM1 gene mutations were more distantly related to the alive outcome. Furthermore, Eigenvector analysis of Pearson correlations demonstrated RAD50, BRCA1, NF1, ATR, and TP53 being clustered with the alive outcome (**Fig 4B**), suggesting that these gene mutations are the most pertinent predictors of the alive outcome for EOC patients.

## Discussion

In this present study, we analyzed 360 EOC cases on the NCI’s TCGA database to identify patterns in mutation spectrum and frequency in relation to the survival outcomes of patients. It should be noted that these EOC cases analyzed presumably received the standard regimen of platinum-based chemotherapy. Current consensus is that HR deficiency is indicative of a prominent phenotype of improved clinical responses of EOC to platinum-based chemotherapy and/or PARP inhibitors. In theory, patients with HR deficient EOC would expect to have notably improved survival. However, our study suggests that other gene mutations should be taken into consideration to predict the survival outcomes of EOC patients.

We demonstrate that mutations in six genes strongly correlated with positive survival were BRCA1, NBN, BRIP1, RAD50, PTEN, and PMS2. Of these genes, BRCA1, NBN, BRIP1, and RAD50 are part of the HR pathway [14–16]. On the other hand, our analysis shows that mutations in six genes strongly correlated with negative survival were FANCE, FOXM1, KRAS, FANCD2, TTN, and CSMD3. Contrary to conventional wisdom, mutations in FA genes FANCE and FANCD2 were strongly correlated with negative survival despite their presumed role in the HR pathway [17, 18]. We speculate that the association of FA genes with HR repair pathway is context-dependent. Future analysis in cancers of other tissue origins will help clarify the perplexed functions of these genes in survival outcomes. The important known functions of some genes possibly associated with the survival outcomes are summarized in **Table 4**.

**Table 4:** Summary of the functions of genes associated with patient survival outcomes.

Gene	FUNTION
**BRCA1**	BRCA1 is involved in the multiple steps of the HR repair pathway. It interacts with the MRN complex to stimulate end resection and interacts with the PALB2-BRCA2-RAD51 complex to promote HR repair. BRCA1 mutations cause HR deficiency. [19–21]
**BCRA2**	BRCA2 regulates RAD51 activity and is indispensable for the HR repair pathway. BRCA2 mutations cause HR deficiency. [22, 23]
**PTEN**	PTEN reduces the intracellular level of phosphatidylinositol 3-phosphate and negatively regulates AKT signaling. PTEN mutations mimic the HR-deficient phenotypes of BRCA mutations. [24–26]
**KMT2C**	KMT2C is a chromatin-modifying protein involved in transcriptional co-activation and histone methylation activity. Knockdown of KMT2C causes decreased expression in several critical DNA damage response and DNA repair genes including BRCA1, BRCA2, ATM, and ATR. [27–29]
**NF1**	NF1 prevents cell overgrowth by turning off the RAS protein that stimulates cell growth and division. NF1 mutations cause uncontrolled cell proliferation. [30, 31]
**RAD50**	RAD50 interacts with MRE11A and NBN to form the MRN complex. The complex binds to DSBs to facilitate end resection for HR and other DSB repair pathways. [32–34]
**MRE11A**	MRE11 interacts with RAD50 and NBN to form the MRN complex. [33, 34]
**NBN**	NBN interacts with MRA11A and RAD50 to form the MRN complex. [33, 34]
**PALB2**	PALB2 serves as an adaptor protein to bridge BRCA1 and BRCA2-RAD51 for nuclear localization and HR repair. Loss of function mutations of PALB2 mimics RAD51 and BRCA2 mutations. [35, 36]
**CDK12**	CDK12 regulates the expression of genes involved in DNA repair. It is a key regulator of cell cycle progression, transcription, and DNA damage response. It plays an indirect role in HR by affecting critical transcription factors of HR genes such as BRCA1 and BRCA2. CDK12-inactivated cancers often resemble BRCA1/2-inactivated cancers characterized by high genomic instability. [37–39]
**BRIP1**	BRIP1 interacts and with BRCA1 and is involved in HR repair. Mutations in BRIP1 increase the risk of breast and ovarian cancers. [40]
**ATM**	ATM is a serine/threonine kinase activated by DSBs and phosphorylates a variety of downstream targets, including H2AX, p53, BRCA1, and CHK2, necessary for checkpoint activation and DSB repair. It mainly responds to DSBs induced by ionizing radiation. [41, 42]
**ATR**	ATR is a serine/threonine kinase activated by single stranded DNA when replication forks stall or DNA repair intermediates occur. It phosphorylates downstream targets, such as Claspin and CHEK1, and regulates the S phase checkpoint to ensure orderly DNA replication. It responds to both SSBs and DSBs. [43, 44]
**FATs**	FAT cadherins are cell adhesion molecules that function at the cell surface to regulate the tumor suppressive Hippo signaling pathway. FAT-dependent regulation of mitochondrial activity is critical for tissue growth. Loss of function facilitates metabolic changes, malformation of lymphatic system, tumorigenesis, and metastasis. [45–47]
**MMR**	MMRs genes including MLH1, MSH2, MSH6 and PMS2 mediate the repair of mismatch DNA. Mutations in MMR genes lead to hypermutated phenotypes and high genomic instability. [48–50]
**FANCD2**	FA genes including FANCA, FANCB, FANCD2, and FANCE play a role in regulating the HR repair pathway. FANCD2 is activated by the FA core complex in response to DNA damage and interacts with BRCA1, RAD51, and BRCA2 to mediate HR repair. [51, 52]
**FANCE**	FANCE is a part of the FA core complex responsible for sensing DNA damage and activating FANCD2. [53, 54]
**FOXM1**	FOXM1 is a proliferation-associated transcription factor responsible for regulation of cell division, self-renewal, and tumorigenesis. [55, 56]
**KRAS**	KRAS is an oncogenic protein in the RAS/MAPK pathway that relays extracellular signals to promote cell growth and proliferation. [57]
**TTN**	TTN serves as a molecular spring that provides the passive elasticity of striated muscle. It is also identified as a structural protein of chromosomes [58]
**CSMD3**	CSMD3 is one of CSMD proteins involved in cell-cell adhesion and expressed primarily in brain. [59]
**MUC16**	MUC16 is a diagnostic biomarker of EOC (CA125) for poor outcomes. It binds to mesothelin to initiate the invasion of tumor cells to the peritoneum. [60]

Based on our logistic regression model, BRCA1 mutation has the strongest impact on increased survival of EOC patients. We also observe that BRCA1 is identified among the positive genes of both uterine and cervical cancer patients. BRCA1 is a tumor suppressor gene which plays a critical role in HR-mediated DNA double strand break (DSB) repair. Our Pearson correlation analysis corroborates that BRCA1 mutation is the most correlative with the alive outcome in EOC patients. However, the positive link between the alive outcome and BRCA2 mutation is obscure. Mutations in BRCA1 and BRCA2 lead to inaccurate repair by nonhomologous end joining (NHEJ), thereby causing genome instability and DNA damaging-induced cell death. Therefore, it is believed that EOC with BRCA1 or BRCA2 mutation is highly responsive to platinum-based chemotherapy and PARP inhibitor therapy. However, our analysis demonstrates that EOC patients with BRCA1 mutation have a higher overall survival rate than BRCA2-mutated EOC cases (72.7% vs 41.2%) (**Table 1**). BRCA1 is involved in the multistep process of HR whereas BRCA2 serves a specific role in the later stage of the HR pathway. Thus, mutations in BRCA1 may have a more profound effect on the HR pathway, thereby translating to favorable prognosis and a high rate of response to platinum-based chemotherapy, compared with BRCA2 mutations. We also speculate that BRCA2-mutated EOC concurrently harbors other mutations in genes associated with decreased survival, such as FAT1, MUC16, and TTN (**Fig 3A**).

A similar study has been conducted using TCGA mutational-signature-based homologous recombination deficiency (HRD) measures, including HRD score and loss of heterozygosity (LOH), to predict clinical responses to platinum/PARP inhibitors and the survival outcomes [13]. It concludes that identifying HRD in EOC patients can accurately predict the long-term survival. Our logistic model in principle corroborates this conclusion because 4 of the 6 positive genes are involved in the HR repair pathway. In contrast, our study additionally takes the negative genes into account to predict the survival outcomes. Therefore, our model not only provides predictive capacities when platinum/PARP inhibitors are properly used, but also helps identify potential new targets (e.g. FOXM1, KRAS, PTEM) for future development of therapeutic strategies [61, 62]. Furthermore, our model offers the flexibility to identify a gene mutation signature/pattern unique to each type of gynecologic cancer patients (**Tables 2, S1 and S3**). It is generally believed that HRD is highly useful to predict the response of EOC to platinum-based chemotherapy and PARP inhibitors (breast cancer to a lesser extent), but less impactful for the treatment of other types of cancers.

Despite the utility of our predictive model across EOC, uterine, and cervical cancer patients, there are weakness and limitations of our study that can be improved in the future investigation. We acknowledge that the accuracy of the predictive model for the survival outcomes of EOC patients was only 64% whereas the accuracy for uterine cancer and cervical cancer patients was 82% and 79%, respectively. This suboptimal accuracy for EOC patients may be attributed to a low number of cases with some gene mutations (less than 10 patients), such as KRAS, NBN, and MRE11A. In addition, EOC is the most lethal gynecologic cancers because it is difficult to diagnose until advanced stage and frequently relapses [1]. Furthermore, some critical confounding factors, including tumor grade/stage, tumor subtype, primary/recurrent disease, treatment type/response, and age of diagnosis, was not included in this study. These shortcomings can be addressed in the future when our analysis on the TCGA database includes more patients and clinical information.

## Supporting information

S1 Fig Performance of logistic regression modeling. (PDF)

S2 Fig Kaplan-Meier survival analysis of 437 uterine cancer and 174 cervical patients. (PDF)

S1 Table Logistic regression analysis of 23 gene mutations and survival outcomes in 437 uterine cancer patients. (PDF)

S2 Table Classification metrics for evaluation of the predictive model for the survival outcomes of uterine cancer patients. (PDF)

S3 Table Logistic regression analysis of 21 gene mutations and survival outcomes in 174 cervical cancer patients. (PDF)

S4 Table Classification metrics for evaluation of the predictive model for the survival outcomes of cervical cancer patients. (PDF)
