# Supplementary material for: Predictive modeling of gene mutations for the survival outcomes of epithelial ovarian cancer patients
Source: PLoS One. 2024 Jul 8;19(7):e0305273. doi: 10.1371/journal.pone.0305273 (PMC11230535; doi:10.1371/journal.pone.0305273)
Supplement: S1 Fig — (PDF) [file pone.0305273.s001.pdf]

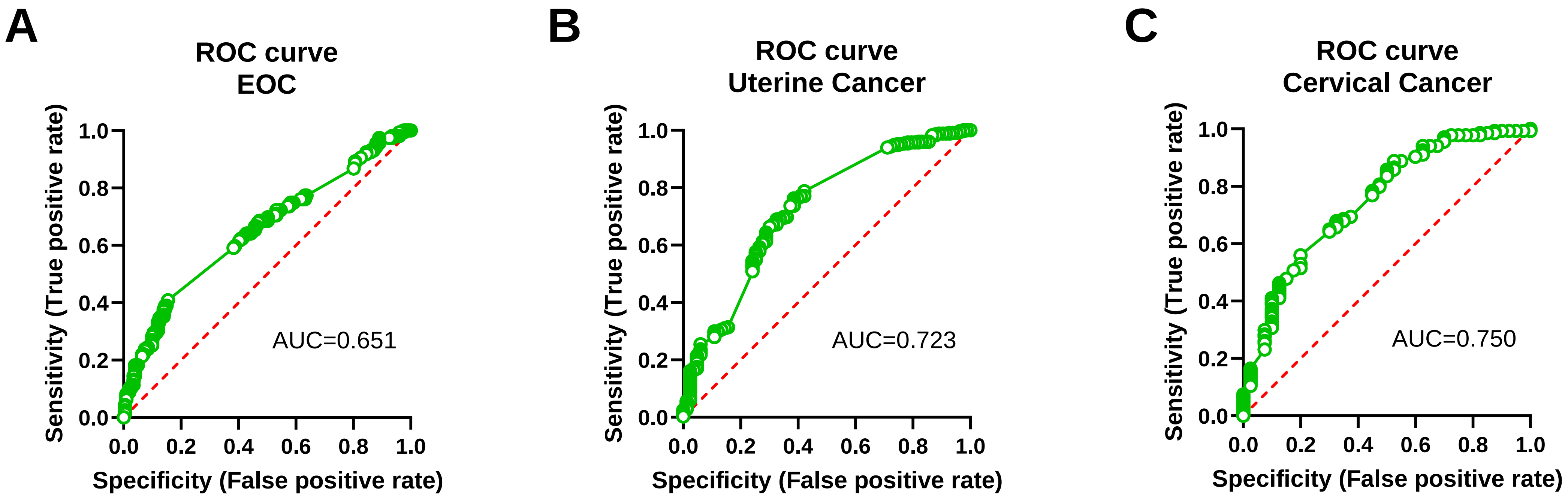

S1 Fig. Performance of logistic regression modeling. The area under the ROC curve is used to evaluate the performance of the logistic regression models for EOC (A), uterine cancer (B), and cervical cancer (C).
