# Supplementary material for: Predictive modeling of gene mutations for the survival outcomes of epithelial ovarian cancer patients
Source: PLoS One. 2024 Jul 8;19(7):e0305273. doi: 10.1371/journal.pone.0305273 (PMC11230535; doi:10.1371/journal.pone.0305273)
Supplement: S2 Fig — (PDF) [file pone.0305273.s002.pdf]

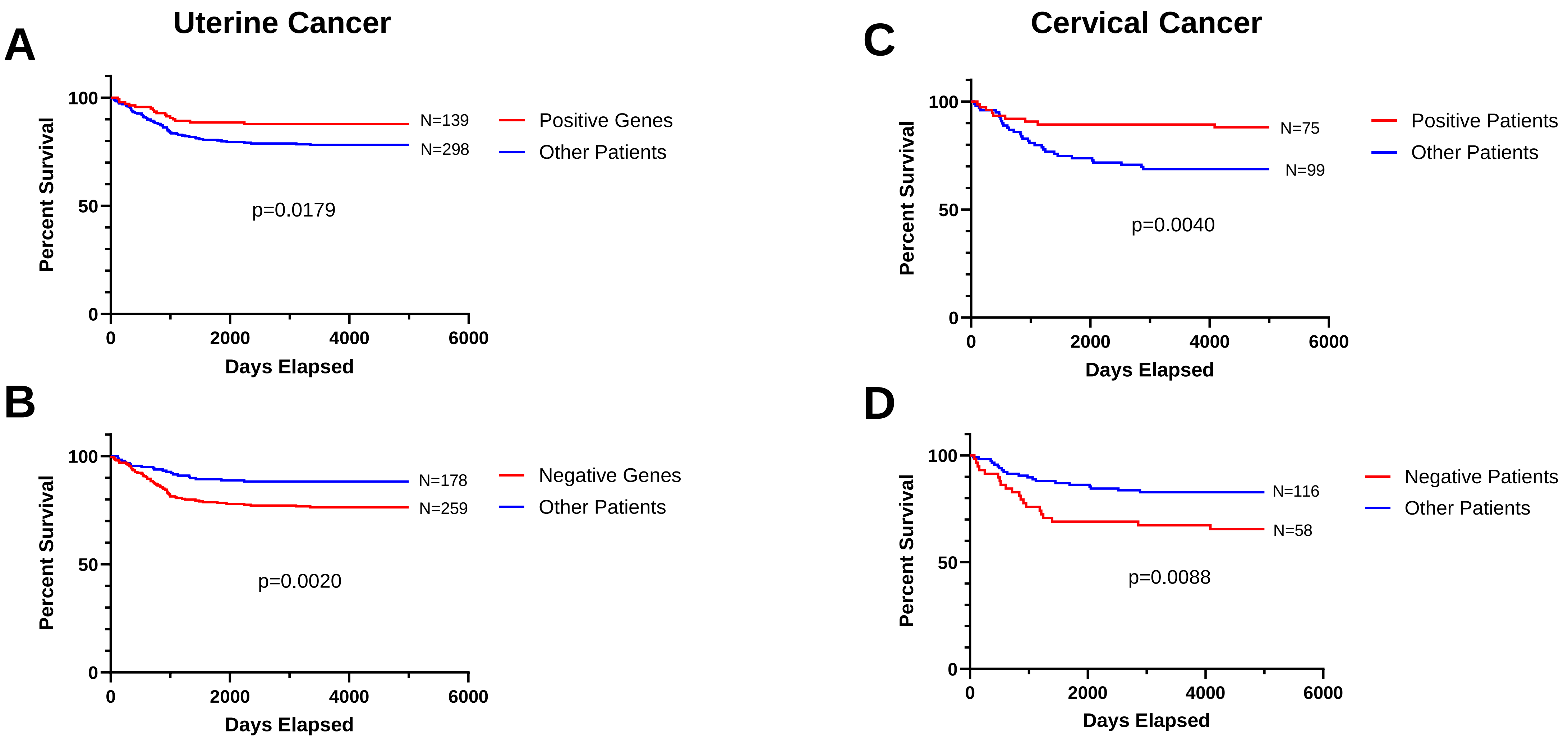

S2 Fig. Kaplan-Meier survival analysis of 437 uterine cancer and 174 cervical patients. Kaplan-Meier survival analysis was performed on the basis of stratified patient populations containing mutations in positive genes (ATM, FANCD2, BRCA1, MSH6, CHEK2, and MUC16) (A), and negative genes (CDK12, BRIP1, FAT3, TP53, NF1, and PMS2) (B) for uterine cancer patients. The same analysis was conducted for cervical cancer patients containing mutations in positive gene (CSMD3, FAT1, BRCA1, PALB2, NF1, and RB1) (C), and negative genes (MLH1, FANCD2, BRCA2, CDK12, TP53, and FAT3) (D). The number of patients stratified (N) was also shown.
