# Supplementary material for: Predictive modeling of gene mutations for the survival outcomes of epithelial ovarian cancer patients
Source: PLoS One. 2024 Jul 8;19(7):e0305273. doi: 10.1371/journal.pone.0305273 (PMC11230535; doi:10.1371/journal.pone.0305273)
Supplement: S1 Table — (PDF) [file pone.0305273.s003.pdf]

S1 Table. Logistic regression analysis of 23 gene mutations and survival outcomes in 491 uterine cancer patients. Blue, positive genes; Red, negative genes.

Variable	Estimate	Estimate 95% CI	Odd ratio	Odd ratio 95% CI
**Intercept**	1.658 ± 0.415	0.8501 to 2.483	5.25	2.340 to 11.97
**ATM**	1.575 ± 0.724	0.298 to 3.200	4.83	1.348 to 24.54
**FANCD2**	1.444 ± 1.327	-0.740 to 4.749	4.24	0.477 to 115.4
**BRCA1**	0.979 ± 1.221	-1.061 to 4.100	2.66	0.346 to 60.36
**MSH6**	0.609 ± 0.852	-0.883 to 2.599	1.84	0.413 to 13.45
**CHEK2**	0.581 ± 1.145	-1.326 to 3.599	1.79	0.266 to 36.58
**MUC16**	0.521 ± 0.408	-0.248 to 1.365	1.68	0.780 to 3.915
**FAT4**	0.512 ± 0.452	-0.332 to 1.454	1.67	0.717 to 4.280
**PTEN**	0.291 ± 0.387	-0.464 to 1.058	1.34	0.629 to 2.879
**FAT1**	0.281 ± 0.466	-0.591 to 1.256	1.32	0.554 to 3.511
**KRAS**	0.236 ± 0.410	-0.537 to 1.083	1.27	0.585 to 2.953
**ATR**	0.212 ± 0.625	-0.952 to 1.542	1.24	0.386 to 4.672
**MLH1**	0.202 ± 0.892	-1.382 to 2.255	1.22	0.251 to 9.536
**KMT2C**	0.068 ± 0.452	-0.778 to 1.011	1.07	0.459 to 2.749
**MSH2**	0.058 ± 1.208	-2.071 to 3.128	1.06	0.126 to 22.83
**NBN**	-0.383 ± 0.985	-2.164 to 1.823	0.68	0.115 to 6.188
**CSMD3**	-0.418 ± 0.356	-1.105 to 0.298	0.66	0.331 to 1.348
**FANCE**	-0.610 ± 1.006	-2.445 to 1.630	0.54	0.0867 to 5.105
**PMS2**	-0.656 ± 0.800	-2.153 to 1.073	0.52	0.116 to 2.925
**NF1**	-0.749 ± 0.531	-1.768 to 0.340	0.47	0.171 to 1.405
**TP53**	-0.785 ± 0.391	-1.552 to -0.0152	0.46	0.212 to 0.985
**FAT3**	-1.031 ± 0.442	-1.890 to -0.144	0.36	0.151 to 0.866
**BRIP1**	-1.277 ± 0.581	-2.418 to -0.106	0.28	0.0891 to 0.899
**CDK12**	-1.711 ± 0.740	-3.188 to -0.236	0.18	0.0413 to 0.790
