# Supplementary material for: Predictive modeling of gene mutations for the survival outcomes of epithelial ovarian cancer patients
Source: PLoS One. 2024 Jul 8;19(7):e0305273. doi: 10.1371/journal.pone.0305273 (PMC11230535; doi:10.1371/journal.pone.0305273)
Supplement: S2 Table — (PDF) [file pone.0305273.s004.pdf]

S2 Table. Classification metrics for evaluation of the predictive model for the survival outcomes of uterine cancer patients.

	Predicted dead (number of patients)	Predicted alive (number of patients)	Accuracy (%)	Precision (%)	Recall (%)
**Actual dead**	6	77	81.5	82.0	98.9
**Actual alive**	4	350			
