# Supplementary material for: Predictive modeling of gene mutations for the survival outcomes of epithelial ovarian cancer patients
Source: PLoS One. 2024 Jul 8;19(7):e0305273. doi: 10.1371/journal.pone.0305273 (PMC11230535; doi:10.1371/journal.pone.0305273)
Supplement: S3 Table — (PDF) [file pone.0305273.s005.pdf]

S3 Table. Logistic regression analysis of 21 gene mutations and survival outcomes in 174 cervical cancer patients. Blue, positive genes; Red, negative genes.

**Variable**	**Estimate**	**Estimate 95% CI**	**Odd ratio**	**Odd Ratio 95% CI**
**CSMD3**	2.678 ± 1.451	0.447 to 6.461	14.55	1.564 to 639.5
**FAT1**	1.385 ± 0.819	-0.0236 to 3.342	4.00	0.977 to 28.28
**Intercept**	1.278 ± 0.372	0.562 to 2.035	3.59	1.754 to 7.649
**BRCA1**	1.276 ± 1.116	-0.540 to 4.264	3.58	0.583 to 71.11
**PALB2**	1.098 ± 1.557	-1.764 to 4.749	3.00	0.171 to 115.4
**NF1**	0.739 ± 0.916	-0.882 to 2.869	2.09	0.414 to 17.62
**RB1**	0.584 ± 0.802	-0.885 to 2.337	1.79	0.413 to 10.35
**BRIP1**	0.322 ± 1.259	-1.933 to 3.449	1.38	0.145 to 31.46
**ATM**	0.285 ± 0.909	-1.434 to 2.246	1.33	0.238 to 9.453
**FAT4**	0.194 ± 0.690	-1.089 to 1.677	1.21	0.336 to 5.349
**PTEN**	0.193 ± 0.654	-1.024 to 1.599	1.21	0.359 to 4.946
**KMT2C**	-0.114 ± 0.459	-1.007 to 0.805	0.89	0.365 to 2.236
**MUC16**	-0.315 ± 0.432	-1.162 to 0.543	0.73	0.313 to 1.720
**KRAS**	-0.462 ± 0.663	-1.741 to 0.901	0.63	0.175 to 2.462
**ATR**	-0.537 ± 0.827	-2.131 to 1.215	0.58	0.119 to 3.371
**NBN**	-0.659 ± 1.382	-3.367 to 2.595	0.52	0.0345 to 13.40
**FAT3**	-0.696 ± 0.732	-2.109 to 0.833	0.50	0.121 to 2.299
**TP53**	-0.728 ± 0.614	-1.928 to 0.521	0.48	0.146 to 1.684
**CDK12**	-0.994 ± 0.657	-2.303 to 0.315	0.37	0.0999 to 1.370
**BRCA2**	-1.137 ± 0.900	-2.938 to 0.702	0.32	0.0530 to 2.017
**FANCD2**	-1.188 ± 1.124	-3.507 to 1.176	0.30	0.0300 to 3.243
**MLH1**	-1.996 ± 1.358	-5.245 to 0.597	0.14	0.00528 to 1.816
