# Supplementary material for: Predictive modeling of gene mutations for the survival outcomes of epithelial ovarian cancer patients
Source: PLoS One. 2024 Jul 8;19(7):e0305273. doi: 10.1371/journal.pone.0305273 (PMC11230535; doi:10.1371/journal.pone.0305273)
Supplement: S4 Table — (PDF) [file pone.0305273.s006.pdf]

S4 Table. Classification metrics for evaluation of the predictive model for the survival outcomes of cervical cancer patients.

	Predicted dead (number of patients)	Predicted alive (number of patients)	Accuracy (%)	Precision (%)	Recall (%)
**Actual dead**	7	33	79.3	79.9	97.8
**Actual alive**	3	131			
